# Implementing a clinical pharmacy service in hematology

**DOI:** 10.1590/S1679-45082016AO3667

**Published:** 2016

**Authors:** Tatiane Fernandes Farias, Karina da Silva Aguiar, Inajara Rotta, Klezia Morais da Silva Belletti, Juliane Carlotto

**Affiliations:** 1 Hospital de Clínicas, Universidade Federal do Paraná, Curitiba, PR, Brazil.

**Keywords:** Drug evaluation, Pharmacy service, hospital, Hematologic diseases, Antineoplastic agents, Hematologic agents, Drug prescriptions

## Abstract

**Objective:**

To implement a clinical pharmacy service focused on the comprehensive review of antineoplastic drugs used in therapy of hematological diseases.

**Methods:**

An interventional study was conducted in a Brazilian tertiary teaching hospital in two different periods, with and without a clinical pharmacy service, respectively. This service consisted of an antineoplastic prescription validation (analysis of patients' characteristics, laboratory tests, compliance with the therapeutic protocol and with pharmacotechnical parameters). When problems were detected, the pharmacist intervened with the physician or another health professional responsible for the patient. Inpatients and outpatients with hematological diseases were included.

**Results:**

We found an increased detection of drug-related problem by 106.5% after implementing the service. Comparing the two periods, an increase in patients' age (26.7 years *versus* 17.6 years), a predominance of outpatients (54% *versus* 38%), and an increase in multiple myeloma (13% *versus* 4%) and non-Hodgkin lymphoma (16% *versus* 3%) was noted. The most commonly found problems were related to dose (33% *versus* 25%) and cycle day (14% *versus* 30%). With regard to clinical impact, the majority had a significant impact (71% *versus* 58%), and in one patient from the second period could have been fatal. The main pharmaceutical interventions were dose adjustment (35% *versus* 25%) and drug withdrawal (33% *versus* 40%).

**Conclusion:**

The pharmacy service contributed to increase the detection and resolution of drug-related problems, and it was an effective method to promote the safe and rational use of antineoplastic drugs.

## INTRODUCTION

Patient safety regarding use of medications has received special attention all over the world through actions to improve the quality of healthcare services.^(1,2)^ Over the last years, the discussion about the occurrence of medication-related-problem (MRP) and their importance as a risk factor of morbidity and mortality has gained notoriety.^(3,4)^

Medication-related-problem in antineoplastic therapy of hematological diseases may produce serious adverse effects, since the drugs used are a part of complex regimens, besides having high toxicity and low therapeutic indexes.^(5,6)^ The treatment of hematological diseases is composed of numerous agents (chemotherapy, support therapy, and medications for comorbidities), and requires intense monitoring, since the medications should be administered in a programmed manner (only on certain days of the week or month), and dose changes are frequent due to alterations in body surface area, clinical status of patients, and development of toxicity (renal, hepatic, hematological, and others).^(7,8)^

Validation of the prescription by clinical pharmacists may identify substances that generate MRP, allowing actions to prevent the occurrence of unfavorable results of drug therapy, and contributing to patient safety and rational pharmacotherapy.^(9)^

In literature, there is divergence among the concepts of clinical pharmacy services (CPS). According to Roberts et al., they are “services offered by pharmacists in which they use their knowledge and expertise in order to improve pharmacotherapy and management of the disease, in face of interaction with the patient or with another healthcare professional, when necessary.”^(10)^ Another definition is given by Gastelurrutia et al.,^(11)^ stating that CPS are “oriented in favor the patient and performed by pharmacists who, requiring specific knowledge, have the objective of improving the process of use of the medications and/or the results of pharmacotherapy.”^(10,11)^

Despite studies reinforcing the importance of CPS with a focus on optimization of antineoplastic therapy, especially in outpatients, there are no studies in Brazil with the purpose of evaluating the impact of a clinical service on detection and resolution of MRP involving antineoplastic therapy in hematological patients.^(12–15)^

## OBJECTIVE

To implement a clinical pharmaceutical service centered on the clinical review of the antineoplastic drugs used to treat hematological diseases, characterizing the most common medication-related problems, including their clinical impact and characteristics of the patients involved, as well as the main pharmaceutical interventions carried out.

## METHODS

An interventional study was conducted in a tertiary university hospital with 406 beds, located in the Southern Region of Brazil, a reference in treatment of hematological neoplasms (*e.g.*, chronic and acute leukemia) and benign diseases (such as severe aplastic anemia, Fanconi's anemia, Wiskott-Aldrich syndrome, among others).

The study was performed in two distinct periods: period A, from November 2012 to November 2013, and period B, from November 2014 to November 2015, based on the absence and presence of a CPS, respectively. The study included inpatients and outpatients with hematological diseases and with prescriptions for antineoplastic medications. This study was approved by the Research Ethics Committee, under protocol number 971.005, CAAE: 41606215.3.0000.0096.

During the first period of the study, the prescription was validated daily by pharmacists and pharmacy residents, considering pharmacotechnical parameters (compatible diluents and infusion volume) and compliance with the medical recommendation regarding the therapeutic scheme used (day of the cycle and dose of the medication), recorded by the prescriber on the hospital's computerized system. The medical recommendation was available for all pediatric patients (both inpatients and outpatients) and for adult inpatients. Hence, the analysis of the treatment regimen was performed only for these groups of patients.

During the second period, a CPS was implemented, and pharmacists and pharmacy residents performed a complete review of all antineoplastic medications prescribed daily for adult and pediatric patients with hematological neoplasms seen at outpatients and inpatients units. The following parameters were evaluated: patient characteristics (primary neoplasm, age and weight or body surface); laboratory tests (checking the need to adjust dose of chemotherapeutical agent, as per result of biochemical and hematological tests); conformity with the therapeutic protocol (diagnosis, number of the cycle, interval, medication dose, support therapy, and order of administration of medications); compliance with pharmacotechnical parameters (compatible diluent, infusion volume, concentration of the solution, physical-chemical and microbiological stability of the compounded medication); and comparison of the medical recommendation recorded in the system, whenever applicable, with the information recommended in the literature.

With the objective of providing support to the development of CPS, two guides were developed, one containing the main therapeutic protocols for treatment of hematological neoplasms and another guide with the standardized dilution of chemotherapy agents. The first, developed in connection with the medical team of the hematology unit of the hospital, contained the standardization of the therapeutic regimens (drugs, doses, numbers of cycles, and interval), support therapy, and the order of administration of the medications. The dilution guide contained the pharmacotechnical aspects of the antineoplastic agents (compatible diluents, infusion volume, solution concentration, and physical-chemical and microbiological stability).

Additionally, during the prescription validation process, the pharmacist had access to the Micromedex Solutions^®^, UptoDate^®^, and MEDLINE^®^ databases. Other sources of information searched included the Brazilian Clinical Oncology Manual, the National Comprehensive Cancer Network, and specific articles of the area.

After detection of MRP, the pharmacists would intervene along with the physician or other healthcare professional responsible for the patient, to establish the best management to be adopted to solve the problem. The necessary data for the analysis of the prescription were obtained by the hospital's internal system, and the information regarding MRP and pharmaceutical interventions (PI) were recorded on an electronic spreadsheet for clinical documentation of the organization activities. The variables collected in this study included age, sex, setting where chemotherapy was prescribed (inpatient or outpatient unit), types of MRP, types of PI, acceptability of the interventions, primary neoplasm, and medications involved in the MRP.

Classification of the MRP and PI was made according to the categories standardized at the organization, which uses the classification of the American Society of Health-System Pharmacists.^(16)^ Assessment of the clinical relevance of the detected MRP was made based on a modified version of the scale by Overhage et al.,^(17)^ in which the MRP are divided into five categories: (1) potentially fatal; (2) serious; (3) significant; (4) mild, and (5) devoid of error directly involved with the patient.^(18)^

To evaluate the impact of the proposed CPS, two periods were compared (A *versus* B) with distinct pharmaceutical services. For this, descriptive statistics were used in order to report percentage, mean, and standard deviation of the categorical and numerical variables, respectively.

For the statistical inference analysis, the Kolmogorov-Smirnov test was used to analyze the distribution of the numerical variables. The χ^2^ and Fisher's tests were used for the categorical variables, and Student's *t* test, for the independent samples. All analyses were two-tailed, and results with a p<0.05 were considered statistically significant.

## RESULTS

In all, more than 13 thousand prescriptions were analyzed during the periods included in this study; in that, 7,894 prescriptions validated during period A and 5,671 prescriptions during period B. A 106.5% increase was noted in the detection of MRP, since in the absence of CPS, 73 were detected, and in the presence of the service, 112 MRP (Table 1).

**Table 1:** Characteristics of patients receiving intervention

Characteristics		Period A n (%)	Period B n (%)	p value
Number of prescriptions		7894	5671	
	MRP	73	112	
Patients				
	Age, mean (SD)	17.6 (13)	26.7 (21.7)	0.031
	Sex (male)	49 (67)	60 (54)	0.067
Setting				
	Outpatient	28 (38)	61 (54)	0.032*
	Inpatient	45 (62)	51 (46)	
Diagnoses				
	Acute lymphoid leukemia	38 (52)	53 (47)	0.53
	Non-Hodgkin lymphoma	2 (3)	18 (16)	<0.001*
	Acute myeloid leukemia	15 (21)	2 (2)	<0.001*
	Multiple myeloma	3 (4)	14 (13)	0.068
	Hodgkin's lymphoma	4 (5)	8 (7)	0.766
	Others	11 (15)	17 (15)	0.012*
Medications				
	Methotrexate	6 (8)	17 (15)	0.16
	Cyclophosphamide	9 (12)	13 (12)	1
	Cytarabine	12 (16)	13 (12)	0.35
	Asparaginase	9 (12)	5 (4)	1
	Filgrastim	1 (1)	9 (8)	0.091
	Vincristine	3 (4)	7 (6)	0.74
	Others	33 (45)	48 (43)	0.75

* p value p<0.05).

MRP: medication-related problem; SD: standard deviation.

Results in absolute and relative (%) numbers, except if specified (mean and standard deviation). Other underlying diagnoses that could have contributed towards increased detection rates for medication-related problem between 2013 and 2015, but that were not included due to their reduced number: chronic myeloid leukemia (zero in 2013 *versus* 5 in 2015) and chronic lymphoid leukemia (zero in 2013, and 2 in 2015).

During period A, a predominance was noted for younger patients relative to period B (17.6 years *versus* 26.7 years) and in the second period, more MRP was observed in outpatients than in inpatients.

As to underlying diseases of patients with MRP, the following hematological neoplasms stand out: non-Hodgkin lymphoma (NHL), multiple myeloma (MM), and acute lymphoid leukemia (ALL). There was a significant increase in the detection of MRP in patients with NHL (p<0.001) and more MRP were detected in patients with MM during period B (p=0.068). On the other hand, during period B, there was a significant reduction in the detection of MRP in patients with acute myeloid leukemia (AML).

The main medications related with MRP were methotrexate, cyclophosphamide, cytarabine, asparaginase, and filgrastim, with no statistically significant difference between the groups.

During period B, the main problems detected were related to dose (n=37), day of the cycle (n=16), duration of treatment (n=14), and incorrect dilution and concentration of the solution (n=14). Whereas during period A, a greater occurrence of MRP due to dose (n=18), day of the cycle (n=22), and omitted medication (n=11) was observed (Table 2).

**Table 2:** Characteristics of detected medication-related problems (n=185)

		Period A n (%)	Period B n (%)
Number of MRP		73 (39)	112 (61)
Type of MRP found			
	Dose	18 (25)	37 (33)
	Day of cycle	22 (30)	16 (14)
	Duration of treatment	4 (5)	14 (13)
	Dilution/concentration of the compounded medication	4 (5)	14 (13)
	Adjustment of dose for laboratory tests	0	7 (6)
	Administration interval	5 (7)	6 (5)
	Medication omitted	11 (15)	6 (5)
	Need to continue treatment	0	5 (4)
	Need for additional medication	0	2 (2)
	Others	9 (12)	5 (4)

MRP: medication-related problem.

**Figure 1:**
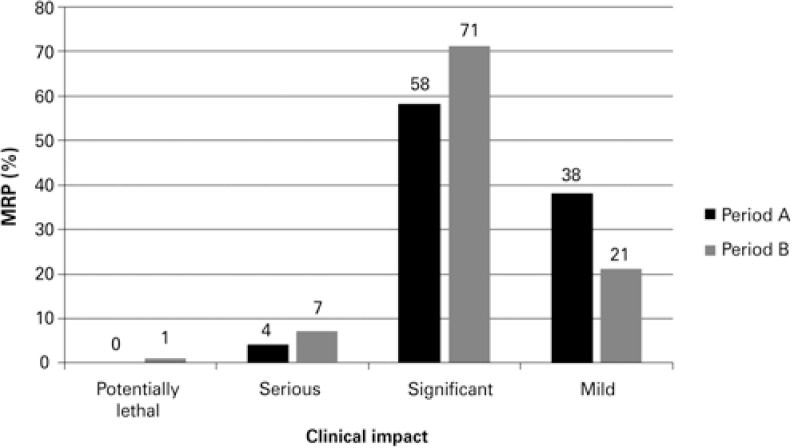
Clinical impact of medication-related problems MRP: medication-related problem.

Most of the MRP in both periods were considered clinically significant (58% in period A, and 71% in period B), and in period B, 7% of the MRP were classified as serious, and 1% was considered as potentially lethal. In period A, only 4% of serious MRP and no potentially lethal MRP were detected (Figure 1).

In both periods of the study (period A and B, respectively), the main PI were involved with dose adjustment (25% *versus* 35%) and withdrawal of medication (40% *versus* 33%). Alteration in the dilution and incorrect concentration of the solution (n=14) and the need to include drug therapy (n=14) were also the most often identified MRP in the second period. Some examples of interventions are presented on table 3. A total of 100% interventions were accepted in period A, and 92%, in period no period B.

**Table 3:** Characteristics of pharmaceutical interventions

Interventions	Period A (n=73) n (%)	Period B (n=112) n (%)	Examples
Dose adjustment	18 (25)	39 (35)	Increase in cytarabine dose (the dose described in the protocol is 3,800mg; the dose prescribed is 38mg); reduction in cyclophosphamide dose (the dose described in the protocol is 900mg; the dose prescribed is 3,100mg).
Suspension of medication	29 (40)	37 (33)	Withdrawal of carboplatin prescribed on the wrong day of the protocol; withdrawal of cyclophosphamide for patient with total bilirubin of >5.0mg/dL; withdrawal of filgrastim for patient with CBC showing leukocyte count >23,390
Alteration of diluent/ concentration of the compounded medications	4 (5)	14 (13)	Alteration of the dilution of rituximab from dextrose to saline, due to incompatibility; increase in volume of saline solution to prepare etoposide, in order to attain the recommended concentration
Inclusion of drug therapy	16 (22)	14 (13)	Inclusion of the drug mesna for patients receiving a high dose of cyclophosphamide, due to the risk of hemorrhagic cystitis; inclusion of MADIT for central nervous system prophylaxis in patient with ALL
Others	6 (8)	8 (7)	Changing frequency of administration of cytarabine (the frequency as per the protocol is every 12 hours, prescribed every 24 hours); alteration of the route of administration of filgrastim (from intravenous to subcutaneous), due to greater comfort for patients

MADIT: methotrexate, cytarabine, and dexamethasone; ALL: acute lymphoid leukemia.

## DISCUSSION

This study compared two distinct periods of validation of medical prescription of antineoplastic agents to treat hematological neoplasms: one period based on a strict analysis of the prescription, and the other with CPS based on a clinical review of the medications. The increase by 106.5% in detection of MRP in the presence of the CPS may be associated with greater safety in the pharmacotherapy prescribed for hematological patients seen at the organization.

The results indicate that during the period in which the CPS was implemented, more MRP were detected in older patients, which might be associated with improvement of the method to detect MRP, for enabling identification of problems in all age groups. In the absence of the service, most MRP were detected in pediatric patients, since the detection method was based on comparison of prescription with the recommendation of the therapeutic regimen recorded in the system, present in all pediatric prescriptions, and only in prescriptions of adult inpatients.

The focus on evaluation of the therapeutic regimen for pediatric patients during the first period of the study may also explain the difference observed in prevalence of diagnoses involved with MRP between the two periods. In period A, the prevalence of ALL was noted, which is a hematological neoplasm with greater incidence in pediatric patients, whereas the presence of CPS allowed the detection of MRP associated to other diagnoses, such as NHL and MM, neoplasms with greater incidence in adult patients, who along with ALL, were the primary diagnoses involved with MRP in the second period.

The high prevalence of MRP in ALL, NHL, and MM may be associated with the complexity of their treatment regimens, which involve a variety of antineoplastic agents at several stages of therapy (induction, consolidation, intensification, or maintenance). Ranchon et al.^(19)^ demonstrated that treatment protocols that involve more than three antineoplastic drugs were related to a greater risk of MRP in prescriptions.

The results also showed a change in treatment setting (outpatient or inpatient unit) of the patient involved with MRP. In the absence of the service, there was a greater occurrence of MRP in hospitalized patients, since for these patients, there is medical recommendation regarding day of cycle and dose of medication in the system. On the other hand, the presence of CPS enabled greater detection of MRP in outpatients, especially adults.

The review of the medications prescribed for the group of patients only started to be performed after implementation of the said service. This fact is very important, because adult outpatients represent the greatest public in terms of treatment of hematological neoplasms at the hospital. White et al. reported that higher rates of errors with antineoplastic medications were found in outpatients, which is consistent with our results.^(20)^

As to the drugs associated with MRP, our results point out that the main drugs were cyclophosphamide, methotrexate, and cytarabine. These medications have a direct relation with the prevalent diagnoses during the periods of the study, since they are part of treatment protocols for the neoplasms discussed. Moreover, the large variety of drugs involved in these complex protocols may lead to a greater incidence of MRP.^(19)^ During the presence of CPS, there was also an increase in MRP with filgrastim, a fact that is related to the inclusion of checking laboratory tests and the observance of the concentrations allowed in diluting the medications.

As to the MRP, the incorrect dose was evident as one of the most frequent problems, and this is the main MRP in the presence of CPS. This result is consistent with other studies conducted, in which the greatest percentage of MRP was dose-associated.^(4,18,21)^

Despite these MRP being grouped in the category of incorrect dose, there was a difference in the profile of errors. During the first period, we noted primarily a discrepancy between the dose indicated in the medical recommendation and the dose effectively prescribed, while in the presence of CPS, incorrect doses were especially detected by analysis of the patient's laboratory tests, and by the verification of conformity of the dose with that recommended in the hospital guidelines.

Checking the antineoplastic dose is extremely important, since attaining the desired therapeutic effect depends on the correct dose, besides avoiding toxicity to the patient, especially after various cycles of chemotherapy in which the patients may have changes in weight, present renal, hepatic, or hematological dysfunction, situations wherein the adjustment of many antineoplastic doses is recommended.

Other common MRP in the presence of CPS were prescriptions on the incorrect day of the cycle, incorrect duration of treatment, and the need to alter the diluent/concentration of the solution of the compounded drug. These errors were detected primarily due to the inclusion of analysis of the therapeutic regimen and of pharmacotechnical aspects.

Compliance with pharmacotechnical parameters is fundamental for reducing incompatibilities, optimizing the administration of antineoplastics, and improving tolerance to these agents, while compliance with the recommended therapeutic regimen leads to the desired clinical response and decreases the risk of toxicity.^(2)^

As to the severity of the MRP, implementing the CPS allowed the detection of a greater number of significant, serious, and potentially lethal MRP. The overdose MRP in one of the cases could have been fatal, considering the dose given was ten-fold higher than that defined in the clinical protocols. In this study, all MRP were detected before reaching the patient.

The main PI performed in both study periods was a request to the prescribing physician to adjust the dose and withdraw the medication, which was directly associated to the MRP detected. A difference was noted in the rate of acceptability between the two periods, since the interventions in period A consisted mainly of adjusting prescription and recommendation, whereas in period B, they were related to the clinical approach.

The interventions not accepted in this period required additional data which was not available to the pharmacy team, such as toxicity not evident on the laboratory tests, which triggered the prescription of under-doses of the antineoplastic agent.

As limitations of this study, we can mention the absence of body surface of the patient in some medical prescriptions, hindering the analysis of the antineoplastic dose. In this way, our dose-ssociated MRP results may be underestimated. Additionally, the patient's medical records were not available electronically, which hindered access to data fundamental for the pharmaceutical validation of the prescription.

## CONCLUSION

The clinical pharmacy services implemented contributed towards increasing the detection of problems related to medications, especially with the dose and with the therapeutic regimen, which, for the most part, presented with a significant clinical impact. This service also afforded the detection of problems related to medications in prescriptions of adult outpatients, especially those undergoing treatment for hematological neoplasms at the hospital.

The clinical pharmacy services proved to be an effective method for guaranteeing patient safety in antineoplastic treatment, providing safe and rational use of the medications.
